# Turning Knowledge Into Action at the Point-of-Care: The Collective Experience of Nurses Facilitating the Implementation of Evidence-Based Practice

**DOI:** 10.1111/wvn.12009

**Published:** 2013-06-24

**Authors:** Elizabeth J Dogherty, Margaret B Harrison, Ian D Graham, Amanda Digel Vandyk, Lisa Keeping-Burke

**Affiliations:** 1 Doctoral Student, School of Nursing, Queen's UniversityKingston, Ontario, Canada; 2 Professor, School of Nursing, Queen's UniversityKingston, Ontario, Canada; 3 Senior Scientist, Centre for Practice-Changing Research, The Ottawa Hospital Research Institute, and Associate Professor, School of Nursing, University of OttawaOttawa, Ontario, Canada; 4 Doctoral Student, School of Nursing, Queen's University, KingstonOntario, Canada; 5 Assistant Professor, Department of Nursing and Health Sciences, University of New BrunswickSaint John, New Brunswick, Canada

**Keywords:** facilitation, evidence-based practice, evidence-informed practice, evidence uptake, knowledge translation, best practice, nursing, critical incident technique, implementation

## Abstract

**Background:** Facilitation is considered a way of enabling clinicians to implement evidence into practice by problem solving and providing support. Practice development is a well-established movement in the United Kingdom that incorporates the use of facilitators, but in Canada, the role is more obtuse. Few investigations have observed the process of facilitation as described by individuals experienced in guideline implementation in North America.

**Aim**To describe the tacit knowledge regarding facilitation embedded in the experiences of nurses implementing evidence into practice.

**Methods:** Twenty nurses from across Canada were purposively selected to attend an interactive knowledge translation symposium to examine what has worked and what has not in implementing evidence in practice. This study is an additional in-depth analysis of data collected at the symposium that focuses on facilitation as an intervention to enhance evidence uptake. Critical incident technique was used to elicit examples to examine the nurses’ facilitation experiences. Participants shared their experiences with one another and completed initial data analysis and coding collaboratively. The data were further thematically analyzed using the qualitative inductive approach of constant comparison.

**Results:** A number of factors emerged at various levels associated with the successes and failures of participants’ efforts to facilitate evidence-based practice. Successful implementation related to: (a) focus on a priority issue, (b) relevant evidence, (c) development of strategic partnerships, (d) the use of multiple strategies to effect change, and (e) facilitator characteristics and approach. Negative factors influencing the process were: (a) poor engagement or ownership, (b) resource deficits, (c) conflict, (d) contextual issues, and (e) lack of evaluation and sustainability.

**Conclusions:** Factors at the individual, environmental, organizational, and cultural level influence facilitation of evidence-based practice in real situations at the point-of-care. With a greater understanding of factors contributing to successful or unsuccessful facilitation, future research should focus on analyzing facilitation interventions tailored to address barriers and enhance facilitators of evidence uptake.

## BACKGROUND

The quest for “best” practices in health care involves implementing available evidence into practice. Despite growing bodies of evidence, moving evidence from peer-reviewed journals and guidelines into practice remains challenging. Efforts have shifted from focusing on methods for rigorously synthesizing study results into practice recommendations and improving guideline quality toward implementation. We are only beginning to recognize processes involved, and many interventions need additional study. There is still limited understanding regarding what approaches are effective in what contexts (Brouwers et al., 2011; Grimshaw et al., 2006; Kitson et al., 2008).

In addition to the quality and nature of the evidence and context, facilitation is considered necessary for enabling successful implementation (Kitson, Harvey, & McCormack, 1998). Facilitation is “the process of enabling (making easier) the implementation of evidence into practice” (Rycroft-Malone, 2004, p. 300) and support to help practitioners change their attitudes and ways of working (Kitson et al., 1998). Facilitation is considered an appointed role (Harvey et al., 2002) and a process involving individuals and groups (Dogherty, Harrison, & Graham, 2012; Dogherty, Harrison, Baker, & Graham, 2010). The concept is described as multifaceted and a team effort as responsibility does not have to rest on one person (e.g., a facilitator), rather facilitation processes can be shared among individuals (Dogherty et al., 2010). Depending on the approach, the process encompasses a range of activities (Dogherty et al., 2012, 2010) from task-oriented to holistic and enabling actions (Harvey et al., 2002). Although the concept is evolving, not one facilitation approach has been found to be universally effective in enhancing evidence-based practice (EBP; Janes, Fox, Lowe, McGilton, & Schindel-Martin, 2009).

Prior investigations examined nurses’ experiences and perceptions of being involved in facilitated practice development (PD) or guideline implementation projects (e.g., Garbett & McCormack, 2001; Pryor & Buzio, 2010; Ruston, 2002; Wallin, Rudberg, & Gunningberg, 2005). Novice facilitators have also documented their experiences and advice on performing this role (Newton, 2003; Robertson, 2009). In the United Kingdom, PD is a well-established movement incorporating the use of appointed facilitators who assist in developing nursing practice and encouraging change (Janes et al., 2009). Practice developers emphasize the benefits of a multidisciplinary approach involving multiple stakeholders (including managers and service users), use of various methodologies for change (e.g., pedagogical, participatory, etc.), and the challenges of measuring PD outcomes (McCormack, Wright, Dewar, Harvey, & Ballantine, 2007a). In Canada, facilitation is more obtuse. There are many individuals in the Canadian health system who may engage in the facilitation process, but it is embedded in their functions as managers, leaders, advanced-practice nurses, or educators. These individuals are not referred to as “facilitators.” Our understanding is that attempts have not been made to harvest their knowledge regarding their point-of-care facilitation experiences. The question remains: What knowledge do these seasoned “facilitators” or others with experience being facilitated have about the role and function they have played in evidence implementation?

We identified two studies examining perspectives and experiences of facilitators involved in specific projects encouraging best practice implementation in North America (Janes et al., 2009; Stetler et al., 2006). Stetler et al. (2006) found that the role involves various behaviors including problem solving, providing support, and developing relationships. Facilitators identified major elements influencing facilitation as “largely relational in nature and intimately connected to the emotionality” (Janes et al., 2009, p. 166) of those who work within the context. Although we are beginning to understand more about facilitation in implementation science, Stetler et al. (2006) concluded that facilitation must be studied more explicitly across different projects and contexts to define its contribution to successful implementation. Further conceptual clarification of facilitation and its elements are also thought to be required (Helfrich et al., 2010; Kitson et al., 2008). The objectives of this inquiry are:

1. To determine how nurses practicing in Canada articulate their role and the roles of others in facilitating EBP.
2. To describe the tacit knowledge regarding facilitation embedded in the activities of nurses experienced in implementing EBP to synthesize their experiences.

Gathering knowledge from experienced individuals involved in implementation across various settings will provide a more clear articulation of facilitation as an intervention in EBP in Canada. This will allow further comparison to studies conducted in other health systems. Eliciting and documenting actual experiences will add to theoretical and interventional research on facilitation and its influence on the use of practice guidelines.

## METHODS

### Design

An interactive pan-Canadian knowledge translation (KT) symposium was held to examine what has worked and what has not in implementing evidence in practice. This was a reflective exercise based on practitioners’ experiences. Two international researchers with substantial experience in KT, facilitation, and professional PD co-led the symposium: Dr. Margaret B. Harrison and Dr. Alison Kitson. Both researchers are nurses and have been greatly involved in guideline development and implementation. The symposium was designed around critical incident technique. This study is an in-depth analysis of data collected at the symposium focused on facilitation elements of process and output.

### Participants

The Canadian colead of the symposium, Dr. Harrison, invited a purposive sample of skilled nurses from several provinces across Canada, who were directly involved in the implementation of EBP, to participate. Individuals with expertise in KT, professional practice, quality of care, PD, research, and implementation science were sought. In addition, the assembly aimed to represent a diverse and representative sample including nurses from across the continuum of care (e.g., acute care, long-term care, community, etc.) that work with different clinical populations (e.g., chronic disease, cancer, etc.). Selected participants nominated other nurses with facilitation experience, thus providing additional study participants.

Potential participants were sent an information sheet and consent form outlining the nature of the research and were invited to attend the symposium that took place in Toronto, Ontario, Canada, in 2009. Participants mailed signed consent forms to investigators prior to the symposium or submitted forms at the beginning of the symposium. The symposium was funded by the Canadian Institutes of Health Research and, in part, by the Canadian Partnership Against Cancer.

### Data Collection

We examined two sources of data for the purposes of this facilitation analysis. Data sources were participant information sheets and critical incident (CI) briefs.

#### Participant information sheets

Prior to the symposium, participants completed a form eliciting demographic information regarding education, employment, and EBP development or implementation experience. Additional questions were asked about environmental factors (e.g., setting and team organization) and personal factors (e.g., knowledge, skills, and attributes) that have helped participants with the facilitation of EBP.

#### Critical incident briefs

Critical incident technique was used to gather data regarding participants’ experiences with facilitating implementation of EBP at the point-of-care. This long-standing technique involves flexible methods for collecting information on human behavior as a means of extracting its potential value in solving practical problems (Flanagan, 1954). It may be used to identify effective or ineffective behaviors in terms of achieving the aims of certain activities (Flanagan, 1954). The incidents can include memorable events, activities, or role behaviors affecting system or process outcomes (Schluter, Seaton, & Chaboyer, 2007). Others have previously used the technique to examine practical knowledge held by expert nurses (Conway, 1998), and it provided an acceptable format for guiding the reflective exercise with this group.

**Figure 1:**
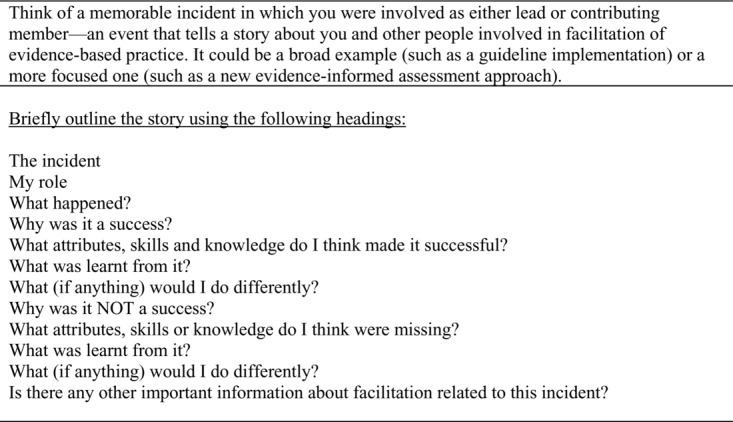
Example of critical incident brief.

Prior to the symposium, each participant (*N* = 20) prepared a written CI brief describing a memorable facilitation experience (see Figure 1). Participants brought completed briefs to the one and a half day symposium. The symposium is best described as an interactive, guided conversation about implementing and facilitating EBP in healthcare contexts. Participants were divided into four groups and shared their facilitation experiences with one another based on their CI briefs. As each participant shared facilitation experiences, the other participants recorded key descriptive words or phrases, both positive and negative, which emerged from the conversations.

### Data Analysis

Descriptive statistics were used to analyze demographic data. To address the first research objective, answers in the CI briefs regarding participants’ roles in the facilitation experiences were analyzed and tabulated (see Figure 1). The CI briefs also addressed the second objective of describing nurses’ tacit knowledge regarding facilitation. The symposium was designed so that participants completed the initial steps of analysis collaboratively during the symposium. As a means of synthesizing their experiences, participants coded raw data (i.e., CI briefs) into positive and negative descriptors and used the descriptors to develop overarching categories related to facilitating EBP.

When analyzing data collected using CI technique, the purpose is to describe and summarize findings so that they may be used in a practical manner (Flanagan, 1954). We analyzed the data further using descriptive content analysis based on the method of constant comparison (Glaser & Strauss, 1967). This approach is used to analyze qualitative data and involves starting with one category and systematically relating it to all other categories such that each category is continuously compared with every other (Glaser & Strauss, 1967). Two analysts uninvolved in organizing the symposium independently compared each category with all other categories identified by participants and synthesized the categories into broader, overarching themes. This process allowed for identification of categories in need of refinement. It is similar to the process identified by Flanagan (1954) for analysis of CIs where incidents, or in this case, descriptors are classified into tentative categories. The tentative categories are then modified or replaced until all descriptors have been classified (Flanagan, 1954). The analysts compared their results using an iterative process and came to consensus on a final set of positive and negative themes and subthemes. An audit trail of the analysis and discussion of emerging themes was documented. Ethics approval was obtained from the Queen's University Health Sciences and Affiliated Teaching Hospitals Research Ethics Board.

## RESULTS

### Participant Characteristics

The symposium brought together a diverse group of 20 nurses from across Canada with various backgrounds and a range of expertise in the development or implementation of EBP (see Table1). Participants had worked, on average, 27 years in nursing and held a combined total of approximately 200 years of EBP experience (range 3–30 years). Both hospital and community healthcare sectors were represented (acute and long-term care), and participants held a range of positions in administration, education, research, and practice with several holding multiple positions simultaneously. Half of the participants were experienced in EBP in the role of project lead. As well, half of the participants indicated that their previous EBP efforts were cross sector, with 90% having been multidisciplinary. Participants worked with a range of clinical populations for whom the evidence or guidelines were implemented (e.g., cancer, wound, critical, palliative care, etc.).

**Table 1:** Participant Demographics

	Total (*n* = 20)
Education and Employment	% (*n*)
Highest level of education in nursing^a^	
• RN diploma	22.2 (4)
• Bachelor's degree	11.1 (2)
• Master's degree	38.9 (7)
• Doctorate (PhD)	27.8 (5)
Current nurse employer	
• Hospital (acute care/complex continuing	
care/rehabilitation/cancer center)	38.9 (7)
• Community (hospice/home care)	22.2 (4)
• College/university	38.9 (7)
• Provincial cancer center/agency	11.1 (2)
Current nursing position^a^	
• Advanced practice nurse, nurse practitioner,	
or clinical nurse specialist	33.3 (6)
• Academic faculty or researcher	22.2 (4)
• Clinical educator	5.6 (1)
• Clinical practice guidelines coordinator	5.6 (1)
• Nurse practitioner/clinical nurse	
specialist/faculty/middle manager	5.6 (1)
• Clinical nurse specialist/faculty/researcher	5.6 (1)
• Faculty/senior manager/staff nurse	5.6 (1)
• Researcher/senior manager	5.6 (1)
Years in nursing – mean (±standard deviation)	26.9 (8.9)
**EBP Development or Implementation**	
**Experience**	**% (*n*)**
Experience in EBP^b^	
• Development	88.9 (16)
• Implementation	88.9 (16)
• Evaluation or monitoring	66.7 (12)
Primary role(s) in EBP^b^	
• Project lead	50.0 (9)
• Researcher	38.9 (7)
• Development team	33.3 (6)
• Implementation team	33.3 (6)
• Professional practice	33.3 (6)
**EBP Development or Implementation**	
**Experience**	**% (*n*)**
• Quality	16.7 (3)
• Administrative director	11.1 (2)
Estimate no. of years involved in EBP – mean (±standard deviation)	10.1 (6.5)

a *n* ≠ 20 due to missing data.

b *n* ≠ 20 as some participants had experience in more than one area of EBP or previously held more than one role in EBP.

### Role Description

When asked about their role in the EBP experiences, most participants identified a specific title (see Table2). Facilitator was the most commonly provided role description. One participant, instead of providing a title, identified activities the role encompassed, namely, working with stakeholders, revising and evaluating the practice protocol, providing education, and building capacity.

**Table 2:** Participants’ Role Descriptions Related to the Critical Incidents

	Total (*n* = 20)
Role^a^	% (*n*)
Facilitator	22.2 (4)
Clinical leader or practice manager	16.7 (3)
Program coleader/associate director of nursing	11.1 (2)
Project lead	11.1 (2)
Researcher	11.1 (2)
Advanced practice nurse	5.6 (1)
Quality coordinator in professional practice in nursing	5.6 (1)

a *n* ≠ 20 due to missing data from four participants and one participant provided a written description as opposed to a role title.

### Positive and Negative Facilitation Experiences: Core Themes

Across the four groups, participants identified a combined total of 590 positive descriptors and 389 negative descriptors. Participants were then divided into two larger groups (one for positive descriptors and one for negative descriptors) where they organized and amalgamated the descriptors into key positive and negative categories. This resulted in a total of 138 positive categories and 86 negative categories representing key concepts related to the facilitation experiences.

Some examples of the experiences participants described in the CI briefs included the implementation of guidelines or protocols, program development and implementation, and introduction of a new model of care. In analyzing the briefs, participants described both successful and unsuccessful experiences in attempting to facilitate EBP in various healthcare contexts. However, in most cases, participants identified what worked and what did not work regardless of whether the implementation was successful or not. In further analyzing and amalgamating the 138 positive categories and 86 negative categories developed by participants based on descriptors from their stories, we identified 10 overarching themes (five positive and five negative) and a number of subthemes representing the key facilitation factors that emerged (see Tables3 and 4). Following this, the raw data, which included the CI briefs and descriptors, were examined to provide descriptive information to support the themes identified below.

**Table 3:** Positive Themes and Subthemes Related to Facilitating Evidence-Based Practice

*Importance of the issue*
• Identifying a high-priority need
• Clinically driven issues based on observed need from practitioners
• Salient and congruent
*Characteristics of the evidence*
• Accessible and easy to use
• Relevant to practice, users, and local context
• Adapted and translated effectively
*Development of partnerships and a project team*
*and engagement of key stakeholders*
• Stakeholder engagement, manager, and frontline buy-in
• Empower those to become involved and overcome barriers
to engagement
• Multidisciplinary project team
• Development of champions
• Strategic partnerships (clinical and academic)
• Shared control and reciprocity
• Lack of conflict of interest
*Strategic process or choreography*
• Strategic plan required in advance
• Ensure resources (e.g., supplies, documentation, and equipment)
• Use of multiple strategies (e.g., education, marketing, etc.)
• Dealing with conflict: diffuse turf issues, negotiation,
and consensus building
• Follow-up, feedback, and celebration: see success that is valued
• Build capacity for sustainability and continued improvement
*Characteristics of the facilitator*
• Clinical and process expert, not necessarily a content expert
• Resource versus authority, mentoring, and coaching
• Ability to broker knowledge, relationships, and support
across levels (e.g., staff and administration)
• Effective communicator, resilient, audacious, visionary,
and passionate about issues
• Possesses interpersonal, relationship, and marketing skills,
authenticity, tenacity, and political savvy

**Table 4:** Negative Themes and Subthemes Related to Facilitating Evidence-Based Practice

*Lack of engagement and ownership*	
• Lack of awareness	
• Lack of interest/seen as not important	
• Lack of buy-in/engagement from stakeholders, physicians,	
multiple sites, project team, clients, and frontline workers	
• Lack of manager involvement and support	
• Passivity/lack of commitment	
*Resource deficits*	
• Lack of access to evidence	
• Lack of equipment and support (e.g., access to	
an advanced practice nurse)	
• Lack of financial and human resources	
• Lack of time	
*Dissonance and conflict*	
• Political and power struggles	
• Failing to bridge differing values/value clash	
• Competing priorities and agendas	
• Fragmentation of the process	
• Disconnect between facilitator and frontline staff	
• Refusal to change	
*Team functioning and workload*	
• Lack of infrastructure/systems	
• Unstable environment, staff turnover, workload issues,	
and burnout	
• Beliefs/attitudes	
• Unsupportive practice context	
• Low morale	
*Lack of evaluation and sustainability*	
• Lack of follow-up	
• Lack of accountability	
• Decreased enthusiasm over time	

**Table 5:** Attributes, Skills, and Knowledge Required for Successful Facilitation

• Ability to engender trust and engage/involve individuals in the change process
• Being respectful, patient, flexible, open-minded, and willing to think “outside” the box
• Having credibility, enthusiasm, a “thick skin,” sense of humor, and a support network
• Skills in organization and administration, teaching, leadership, team building, negotiation and mediation, and “critical research consumption”

### Positive Implementation Factors

#### Importance of the issue

Participants articulated the importance of identifying a clinical practice issue that was “clearly related to a need articulated by the nurses.” This theme originated from descriptors including “asking them what the issues were” and identifying something “that is concrete and real in their everyday practice.” One participant found that nurses who identified the need were more motivated to participate in the implementation, whereas another found that identifying a priority issue (“high volume or high cost”) assisted with stakeholder buy-in.

#### Characteristics of the evidence

One key activity associated with facilitating EBP was searching for evidence. Alternatively, participants described the value of having access to librarian support to assist with searching for evidence and more effectively accessing available literature. Descriptors for characteristics of the evidence included “strong evidence” that was “easy to use.” It was also apparent that groups did not need to start “from scratch” and that previously developed guidelines were utilized or adapted. One participant stated that developing a program based on evidence was “what drove the success” and “a sound protocol is a necessary ingredient for successful implementation.”

#### Development of partnerships and a project team and engagement of key stakeholders

Most participants indicated that their EBP development or implementation experiences involved multidisciplinary efforts. This highlights the value of “careful composition” of the project team including all disciplines involved as well as key stakeholders such as patients and potential end-knowledge users. Participants saw development of academic and clinical partnerships as important along with support from senior leadership and administration. Part of the role was seen as interfacing with frontline staff and stakeholders to engage them in the process. A key theme that many participants mentioned was involving frontline staff in design and delivery of the implementation as a means for empowering them to drive change. One participant stated: “Truly believe in the power of those who will be affected. They need to be in on the process and engaged early on.” One nurse indicated that when frontline workers took ownership of the guideline, implementation progressed more quickly.

#### Strategic process and choreography

This theme relates to the actual implementation process beginning with developing an action plan, including specific timelines. A significant challenge for several participants was ensuring that the right supplies were available such as equipment and personnel. Many participants described the usefulness of conducting a barriers assessment up front to identify potential challenges and develop strategies to overcome them.

A fundamental component of facilitation was use of multiple strategies. One participant advised that “what works with one group may not work with another” and similarly, facilitation techniques that seemed to be effective at the outset of a project may have been less effective over time. Dealing with conflict was something many participants experienced, and in these situations, “not personalizing the issues” and remaining “focused on the challenges” to work through them seemed to be helpful. One participant maintained that “if toxic relational and power dynamics are not dealt with, practice excellence will never be realized.” Participants saw evaluation (structure, process, and outcome) as an important process element but that evaluation “is only useful if we use what is learned from it.”

#### Characteristics of the facilitator

Many facilitator characteristics were found in participants’ descriptors; however, characteristics reflected in Table3 represent the skills and characteristics participants synthesized into categories. Participants stressed the importance of having theoretical and experiential knowledge of EBP and guideline development and implementation, including how to locate sources of evidence, conduct data collection, and critically appraise literature. One participant described having “the comfort to let go and guide versus drive the process” and another became the project's “cheerleader” providing reassurance to the group that the challenges they encountered were not unusual and were just part of the implementation process.

Participants were asked to identify the attributes, skills, and knowledge contributing to successful implementation. Analysis of participants’ responses to this specific question resulted in a number of facilitation skills associated with effectiveness (see Table5) in addition to those listed in Table3, which were identified from participants’ descriptors. These skills included the ability to be flexible and think “outside the box.”

### Negative Implementation Factors

#### Lack of engagement and ownership

This theme relates to staff having a lack of interest in the practice issue and a lack of buy-in and support from stakeholders, staff, and management. Descriptors included: “no physician to support initiative,” “little incentive to change,” “failure to engage key stakeholders,” and “not adopted by organization as ‘their’ project.” Participants’ CI briefs indicated that implementation efforts were compromised significantly when there was lack of management or leadership support for the change and when all individuals affected were not involved from the beginning of the implementation.

#### Resource deficits

Lack of resources was a common issue noted across participants’ facilitation experiences. These resources included but are not limited to: human, financial, access, equipment, time, and evidence. There are costs associated with change. Implementation can take a significant amount of time and one participant described “turmoil” near the end of the project due to a lack of ongoing funding. The limited number of staff and, in particular, access to advanced practice nurses were also a concern. In one case, one participant posited that implementation was unsuccessful due to the clinical nurse specialist having spent little time with staff. While “she could have been a great asset, she was not able to facilitate implementation with those who needed it.” Multiple participants also noted the need for more time, but as one participant asked: “Do we ever have that luxury?”

#### Dissonance and conflict

Several participants described political and power struggles among individuals involved in the implementation. In some cases, there was animosity between different disciplines creating conflict. One participant described “fierce turf wars” generated between disciplines related to who was responsible for performing what procedure within the guideline. Another participant reported that implementation was unsuccessful due to many other changes the organization was trying to implement simultaneously. Multiple and competing priorities resulting in not enough time to focus adequately on implementation were common issues encountered by all participants.

#### Team functioning and workload

One participant described how staff were resistant to change and how the project “brought to light all of the dysfunctional relational dynamics on the unit.” Staff blamed the implementation while the participant maintained that the issues were already there, and it was the implementation that brought them “out of the closet.” Other reasons for many unsuccessful facilitation experiences described by participants related to time and workload. The staff were not able to take time to learn the protocols, and there was no time to implement the recommendations even when the activities were part of daily assessments. Increased workload compromised staff members’ ability to adopt any new activities.

Individuals involved in the implementation who exhibited negative attitudes also affected the process. One participant described how individuals objected strongly to the use of the word “guideline.” When one individual discovered that the group was creating a guideline, that person refused to be associated with the project. This resulted in “tainted relations” for the remainder of the implementation.

#### Lack of evaluation and sustainability

This theme developed from descriptors including: “staff need to see effect” and the “long process takes energy out.” What was missing in several instances was a lack of follow-up or, in other cases, inappropriate outcome measures to evaluate implementation. Commitment and enthusiasm generated by the project team was associated with successful implementation in some cases, but sometimes initial interest was not sustained.

## DISCUSSION

The symposium brought together 20 nurses from across Canada possessing nearly 200 years of collective EBP experience. Participants openly shared both successful and unsuccessful implementation experiences and described their roles differently in relation to each situation. Despite the incidents being different in terms of their focus, clinical specialty, individuals’ roles, and the facilitation processes involved, participants identified a number of common issues. Based on the analysis of descriptors and categories from their stories, we developed 10 themes representing the positive and negative aspects of facilitating EBP. Successful implementation is associated with focus on a priority issue, relevant and easy to use evidence, development of strategic partnerships and a multidisciplinary project team including key stakeholders, use of multiple strategies, and facilitator characteristics and approach. Negative factors influencing facilitation related to missing elements such as lack of engagement, ownership, and resources, in addition to conflict, team functioning and workload issues, and lack of follow-up and sustainability.

Similar to Janes et al.'s (2009) findings, participants described their roles in facilitation using various titles ranging from “guidelines coordinator” to “educator” to “advanced-practice nurse.” Only four participants referred to their role as “facilitators.” Likewise, facilitation is described in the literature as both an appointed role (Harvey et al., 2002) and a process that may involve a number of individuals (i.e., it does not necessarily have to be a “facilitator”; others can take on facilitative activities as part of other roles; Dogherty et al., 2012, 2010). Definitions of facilitation include: the process of making implementation of evidence easier (Harvey et al., 2002) and a “process of *interactive problem solving* and *support*” occurring within “a supportive interpersonal relationship” (Stetler et al., 2006, paragraph 4). As the concept evolves, a more operational definition may be needed to tease out the process of facilitation occurring under the guise of other role descriptions. Without it, there will continue to be a blurring of boundaries and tasks making it difficult to determine what constitutes facilitation as opposed to other activities within other roles.

Participants described a characteristic of facilitators as being a resource as opposed to an authority, which is consistent with earlier descriptions of the role as enabling rather than directive or persuading (Harvey et al., 2002). Participants also indicated that facilitation involves the use of multiple change strategies. Similarly, when Stetler et al. (2006) interviewed facilitators regarding their experiences, they discovered that facilitators were likely to integrate other implementation interventions (e.g., education), and in this way, facilitation is more flexible than other change agent roles.

The use of multiple strategies requires a variety of skills. Our findings indicate that a range of skills, knowledge, and attributes are required for successful facilitation, which is consistent with the literature (Dogherty et al., 2012, 2010; Harvey et al., 2002; Janes et al., 2009; McCormack et al., 2007a; Stetler et al., 2006). Flexibility and credibility are key attributes highlighted in previous work and mentioned as important by participants. However, tenacity was emphasized more frequently as a personal factor that helped participants with facilitation. This indicates that implementation is complex and that persistence to keep the process moving, despite barriers encountered, may be just as important as maintaining flexibility in approaches selected to promote change.

Many of the themes and subthemes reflect findings of other national and international studies where factors influencing facilitation outcomes and general factors influencing PD and implementation of EBP were explored (Janes et al., 2009; McCormack, Wright, Dewar, Harvey, & Ballantine, 2007b; Ploeg, Davies, Edwards, Gifford, & Miller, 2007; Rycroft-Malone et al., 2004). These factors are present at individual, environmental, organizational, and cultural levels. Specifically, the importance and relevance of the issue in addition to development of partnerships with leadership support, key stakeholder engagement, and buy-in continue to be emphasized as important elements for success. In a realist synthesis of findings from interviews with individuals involved in PD initiatives, McCormack et al. (2007a) noted the importance of clinical-academic partnerships and the need to involve service users in PD work. Participants in our study echoed these sentiments but did not provide recommendations for how to enact these partnerships. Similar to McCormack et al.'s findings, the processes to identify and involve stakeholders were not mentioned.

Alternatively, oppositional staff attitudes and beliefs and time and resource deficits associated with workload and staff shortages were hindrances to implementation consistent with the literature. An emphasis on evaluation, follow-up, and sustainability emerged as key factors influencing success. Enthusiasm may wane over time; therefore, staff must “see success that is valued” and capacity built into health systems for continued improvement. The fact that these elements were key issues highlights practitioners’ increasing concern that evidence uptake may not be sustained. It also reflects increasing interest in the field regarding identifying mechanisms for sustainability. This may also explain why participants emphasized tenacity as an essential characteristic of effective facilitators.

### Implications

Participants identified common pitfalls and successes in their facilitation experiences in implementing EBP. These findings are useful for practitioners and organizations in planning for change. Certain factors could be capitalized on for success and plans made to address barriers that may be encountered. The factors identified indicate that facilitation of EBP encompasses activities across the spectrum of well-described phases included in planned action theories. Graham et al. (2007) conducted a review of 31 planned action theories, and participants’ experiences align with many elements included in these theories such as identifying a relevant practice issue for change, adapting evidence to the local setting, assessing barriers, implementing interventions, and tailoring strategies to the local context with an emphasis on evaluating and sustaining knowledge use. The results of this study lend support to planned action elements of these theories as existing in the clinical setting.

According to Kitson et al. (1998), successful implementation of evidence into practice is dependent on the relationship between evidence, context, and facilitation. Participants in this study identified various factors, both helping and hindering implementation, related to evidence (e.g., relevance, ease of use, etc.) and context (e.g., leadership support, engagement, infrastructure, etc.). Interestingly, only one of the positive themes related to context (development of partnerships and engaging key stakeholders) while nearly all negative themes, aside from evaluation, were associated with context. As such, context is an influential factor and something to assess and consider throughout implementation. Practice developers positioned within an organization are important in terms of overcoming contextual barriers (McCormack et al., 2007b). Recognizing that contextual factors were present throughout the facilitation process, a better approach might be to assess and plan how to address these issues in advance. This is the approach proposed by Kitson et al. (2008) who hypothesize that facilitation may be more effective after a diagnosis of context and an assessment of clinicians’ perceptions of the evidence and patient preferences have taken place. This group is currently investigating tools to assist with these evaluations (Kitson et al., 2008; McCormack, McCarthy, Wright, Slater, & Coffey, 2009), and a facilitation approach is then selected based on this assessment.

Details on successes and failures of participants’ efforts that were systematically analyzed, particularly the skills required, could be of importance in planning education and mentorship programs for individuals whose role involves facilitation. Support is needed to prepare individuals who function as facilitators, whether as a dedicated role or part of another function (e.g., clinical educator). Working with these characteristics may help organizations establish who is a good candidate for this function and determine how to integrate this capacity into existing roles.

Organizations also need to consider resources required for EBP as resource deficits are detrimental to the success of facilitation efforts. Resources include financial, personnel, equipment, support, access to evidence, and time. As Stetler et al. (2006) note regarding lack of protected time, resolving this issue is not straightforward, and implying that making protected time available for facilitation will result in success is naïve. The authors also state that protected time for facilitators may be related to the degree of organizational investment and support for projects. Similarly, perhaps, simply increasing the amount of resources may not be the best or only way to ensure successful facilitation. Resources are scarce in current healthcare systems, and organizations are looking to implement evidence efficiently and effectively. It is important that organizations examine existing resources that could be utilized to promote change (e.g., standing staff development and education sessions, access to library resources, etc.). This will be different across organizations, but it is important that staff input is gathered. It is frontline clinicians who are often most affected by practice change, and they may have ideas on how existing resources could be mobilized. Staff could also indicate what resources, if taken away and reassigned to implementation projects, would be detrimental to everyday practice.

### Limitations

Study results should be interpreted in light of several features. The data source was participants’ reflections, which could potentially cause recall bias or failure to disclose. Participants consisted of researchers and clinicians and were purposively selected from a range of healthcare settings, which may inform transferability of findings. Alternatively, these individuals may have been enthusiastic and supportive of EBP resulting in positive bias. However, in analyzing CI briefs, participants offered balanced perspectives (both positive and negative experiences). Participants were involved in data analysis up front, developing and validating emerging categories based on their descriptors, which ensured that categories described the sampled incidents. Two researchers independently conducted subsequent analysis. Themes were developed from participants’ categories, and an audit trail of the analysis and emerging themes was kept to promote dependability and confirmability.

## CONCLUSIONS

The symposium provided a vehicle for experienced individuals working in KT across the health system to share their facilitation experiences in a structured way, uncovering their experiential learning. Participants engaged in frank discussions regarding complexities involved in facilitating EBP. As opposed to shedding light on actual facilitation activities, participants described the actual, practical experience of being facilitated and doing facilitation in real situations. With greater understanding of factors contributing to successful or unsuccessful facilitation, future research should focus on analyzing facilitation interventions tailored to address barriers and enhance facilitators of EBP. The practical knowledge of what comprises facilitation is essential, including what works under what circumstances. This study has taken a step in this regard by analyzing tacit knowledge of experienced facilitators and those with experience being facilitated from across Canada. Having more clearly articulated the construct, we are better placed to advance both Canadian and international efforts in the uptake of evidence in practice.

## Author information

Elizabeth J. Dogherty, Doctoral Student, School of Nursing, Queen's University, Kingston, Ontario, Canada; Margaret B. Harrison, Professor, School of Nursing, Queen's University, Kingston, Ontario, Canada; Ian D. Graham, Senior Scientist, Centre for Practice-Changing Research, The Ottawa Hospital Research Institute, and Associate Professor, School of Nursing, University of Ottawa, Ottawa, Ontario, Canada; Amanda Digel Vandyk, Doctoral Student, School of Nursing, Queen's University, Kingston, Ontario, Canada; Lisa Keeping-Burke, Assistant Professor, Department of Nursing and Health Sciences, University of New Brunswick, Saint John, New Brunswick, Canada.

Elizabeth J. Dogherty designed and conducted the facilitation analysis from in-depth information that resulted from a symposium where experts reflected on their experiences implementing EBP. She developed the initial manuscript. Margaret B. Harrison, Ian D. Graham, and Lisa Keeping-Burke contributed to the analysis and synopsis of findings. AmandaDigel Vandyk contributed to the data analysis and interpretation. All provided editorial contributions and approved the final version of the manuscript.

The authors would like to extend special acknowledgment to Professor Alison Kitson (University of Adelaide) for her leadership in planning, developing, and coleading the Canadian symposium with Margaret B. Harrison. Also, they gratefully acknowledge the involvement of all participants and others involved in coordinating and assisting with the symposium.

Funding for the symposium was provided by the Canadian Institutes of Health Research (CIHR FRN #88282) and the Canadian Partnership Against Cancer.

Elizabeth J. Dogherty was also supported in part during this work by a CIHR Doctoral Research Award (201010GSD- 249765-194335), a Knowledge Translation Canada Strategic Training Initiative in Health Research (STIHR) Fellowship, and an Ontario Graduate Scholarship.
